# The Impact of Education, COVID-19 and Risk Factors on the Quality of Life in Patients with Type 2 Diabetes

**DOI:** 10.3390/ijerph18052332

**Published:** 2021-02-27

**Authors:** Zvjezdana Gvozdanović, Nikolina Farčić, Hrvoje Šimić, Vikica Buljanović, Lea Gvozdanović, Sven Katalinić, Stana Pačarić, Domagoj Gvozdanović, Željka Dujmić, Blaženka Miškić, Ivana Barać, Nada Prlić

**Affiliations:** 1 General Hospital Našice, Našice 31 500, Croatia; zvjezdana.gvozdanovic@obnasice.hr (Z.G.); hrvoje.simic@obnasice.hr (H.Š.); vikica.buljanovic@obnasice.hr (V.B.); 2 Faculty of Medicine, Josip Juraj Strossmayer University of Osijek, Osijek 31 000, Croatia; lgvozdanovic@mefos.hr (L.G.); skatalinic@mefos.hr (S.K.); pacaric.stana@kbco.hr (S.P.); 3 Nursing Institute “Professor Radivoje Radić”, Faculty of Dental Medicine and Health Osijek Josip Juraj Strossmayer University of Osijek, Osijek 31 000, Croatia; dgvozdanovic@fdmz.hr (D.G.); zeljka.dujmic@bolnicasb.hr (Ž.D.); blazenka.miskic@bolnicasb.hr (B.M.); ivana.barac@fdmz.hr (I.B.); nadaprlic26@gmail.com (N.P.); 4 Department of Surgery, University Hospital Centre Osijek, Osijek 31 000, Croatia; 5 General Hospital “Dr. Josip Benčević” Slavonski Brod, Slavonski Brod 35 000, Croatia

**Keywords:** diabetes mellitus, quality of life, education, coronavirus disease 2019 (COVID-19)

## Abstract

Background: The aim of this study was to examine the impact of education, coronavirus disease 2019 (COVID-19), and risk factors on the quality of life in patients with type 2 diabetes. Methods: A prospective study was conducted in three phases: before education, after education, and in the period of pandemic coronavirus disease 2019 (COVID-19). The subjects were diabetics on oral therapy. To determine the quality of life index, a standardized Ferrans and Powers survey questionnaire was used. Results: A total of 205 participants took part in the study, of which 111 (54.1%) were men and 94 (46%) women. Participants were enrolled in the study between January 2019 and September 2020. Glycated hemoglobin values were significantly higher before education compared to post-education and at the time of COVID-19 (Friedman test, *p* = 0.002), and body mass index was significantly lower after education compared to values before education (Friedman test, *p* = 0.008). The quality of life was significantly lower in all domains in the COVID-19 period (Friedman test, *p* < 0.001). Conclusions: A significant predictor of worse assessment of overall quality of life was male gender and rural place of residence. Disease duration of up to 5 years was a significant predictor of worse assessment in the psychological/spiritual domain, while being married was a predictor of better assessment of the quality of life in the family domain. The education of diabetics brought an increase in the health and quality of life while the coronavirus disease pandemic had negative consequences on the same parameters. We consider it necessary to systematically educate diabetics about the comorbidity of COVID-19.

## 1. Introduction

Diabetes mellitus is one of the fastest growing health challenges of the 21st century, with the number of adults living with diabetes having more than tripled over the past 20 years [1]. Over the last 20 years, a conceptual transformation in the principles of management of type 2 diabetes mellitus (T2DM) has occurred. Treatment for T2DM involves controlling glycemic and metabolic levels, helping patients and their families to adapt their situation on a psychosocial level, preventing serious or chronic complications, decreasing health care costs, ensuring that medications are taken on a regular basis, and, in particular, promoting a change in lifestyle [2]. Chronic non-communicable diseases represent a major public health problem, but today, COVID-19 has become a crucial worldwide health problem [3].

According to estimates by the International Diabetes Federation in 2019, approximately 463 million adults were living with diabetes mellitus; by 2045 this will rise to 700 million. The prevalence of diabetes mellitus in the age group of 20–70 in 2019 was 8.3%, and it is predicted to be 9.6% in 2045. In 2019, a total of 59 million adults in Europe have been diagnosed with diabetes mellitus. It is predicted that, in the period until 2045, the number of patients will increase to 68 million. The prevalence of diabetes mellitus in the age group of 20–79 in Europe in 2019 was 6.3%, and it is predicted that in 2045 it will amount to 7.8%. According to the Croatian National Diabetes Registry, in 2019 in Croatia, 315,298 adults have been diagnosed with diabetes mellitus, and the number of patients is increasing from year to year. Previous research shows that, in Croatia, only 60% of patients have a diagnosis, so it is estimated that the total number of patients is more than 500,000 adults [4].

The data show how important it is to recognize, treat diabetes mellitus in time, and also raise the awareness of the general public to prevent complications and increase the quality of life [1]. The goal of diabetes treatment is the prevention of the onset and progression of micro- and macrovascular complications as well as the achievement of quality of life (QOL) and longevity equivalent to people without diabetes [5].

In the treatment of diabetes mellitus, it is necessary to include patients’ family. Family members need to know what to do in the event of extremely low or high blood glucose. Also, in addition to drug therapy, changes in life habits are needed, so the family’s support and quality communication significantly contributes to the quality of treatment and reduced risk of complications [4].

Education is the process of providing knowledge and skills needed to perform self-care, manage crises, and make lifestyle changes [6,7,8,9,10,11]. A systematic health education model that focuses on factors influencing health behavior in the whole course of the disease is essential. It is based on the relationship between a health educator and a patient, which is particularly appropriate for chronic diseases [10]. While planning a health education program, it is necessary to evaluate what content should be included by considering the needs of the people who will be educated [12]. Investments in diabetes education and prevention are expected to save money in the long term and improve the quality of life of patients with diabetes [12]. Diabetes educators are one of the contributors in providing individualized education and promoting behavior change to enable patients to manage daily and future challenges [10,11,12,13,14,15,16]. The role of a diabetes nurse specialist is an integral part of education [13,14]. Outcomes are largely dependent on decisions made by the patients. A healthy lifestyle that includes good eating habits and physical activity are important because they can be protective factors for weight gain and glycemic control during the COVID-19 era, and education can affect this [17].

Due to the strong human-to-human transmission power of COVID-19, it has become a worldwide pandemic [18]. Clinical studies of patients with COVID-19 have found diabetes to be a major risk factor for disease severity and mortality [18,19,20]. Understanding these risks and the best way to mitigate them in the short and longer term is the key to facilitating informed decision-making during and after the COVID-19 pandemic [21]. The COVID-19 lockdown produced behavioral, psychosocial, and environmental changes which, through a variety of mechanisms, have led to widespread rapid weight gain amongst certain populations worldwide. Khan et al. have termed this phenomenon “covibesity”. There has been an increase in food shopping, food take-a-ways, and an increase in alcohol sales. Furthermore, the combination of working from home, online education, and social media usage have all caused screen time to surge. The food industry has intensified online advertising focused on children. A swift response is needed from all stakeholders to prevent covibesity from becoming a pandemic [22].

The aim of this study was to examine the impact of education, COVID-19, and risk factors on the quality of life in patients with type 2 diabetes.

## 2. Materials and Methods

A prospective study was conducted to determine the quality of life index using a standardized Ferrans and Powers questionnaire and was conducted through three phases. The first phase was from January to September 2019, the second phase was conducted four months after the first phase, and the third phase was conducted in July 2020 during the coronavirus pandemic. The details of the educational program have been published previously [11]. Briefly, the diabetes education was based on the pre-test of diabetic patients and literature on diabetes mellitus [11,23,24]. The educational program was administrated by a team of diabetes specialist nurses, internal specialists, and diabetes experts. The education was conducted individually and in small groups. The group received the required education within five two-hour sessions in groups of 3–4 individuals, during the period of one week, and one individual session [11]. During the educational program, health parameters of participants were quantified (glucose test, glycated hemoglobin, body mass index, and blood pressure) and they were sent a questionnaire about the quality of life index by Carol E. Ferrans and Marjorie J. Powers. Four months after the education (Phase 2), subjects completed the quality of life test again using the same questionnaire, and measurements were repeated. Four months after (July 2020) the start of the coronavirus disease pandemic, subjects were contacted by telephone to attend a follow up meeting where they completed the questionnaire a third time, and the measurements were repeated (Phase 3). The study was conducted at the Diabetes Clinic of the General County Hospital, Našice, Croatia. The Croatian healthcare system covers all costs of the educational program, and it is available to all. All participants with type II diabetes who came to the diabetes clinic, and who signed informed consent to participate in the study, were included in the research.

The inclusion criteria were male or female patients with type II diabetes who were taking oral hypoglycemics, having at least one treatment prior to the study, with an active record in the diabetes clinic.

The exclusion criteria were patients under 18 and/or over 75 years of age, cognitive and/or mental diseases, illiteracy, patients with a life expectancy of less than 2 years, and patients who did not participate in educational sessions (less than 60% of the sessions). Participants were recruited even if they had previously attended similar educational programs; consequently, we have only one group of respondents and no control group.

**Figure 1:**
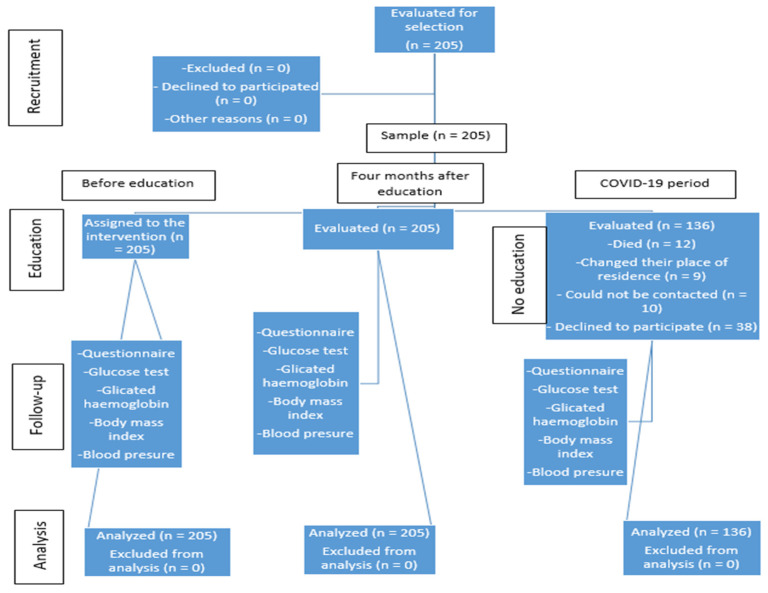
Flow chart of the intervention, participants, and measurements.

In total, 205 patients participated in the first two phases; in the third phase, two thirds of patients participated, a total of 136 (66%). Of the original subjects, 12 (6%) died, 9 (4%) changed their place of residence, 10 (5%) could not be contacted, and 38 (18%) declined to participate in the follow-up, mostly because of the COVID-19 pandemic (Figure 1).

### 2.1. Study Tools

To collect basic data, a survey questionnaire was constructed that provided data on gender, age, level of education, place of residence, marital status, minor children in the family, family members over the age of 60, total disease duration, and the health parameters: fasting blood glucose, glycated hemoglobin, body mass index, and blood pressure. Regarding the COVID-19 pandemic, they were asked how much the pandemic affected them, which precautionary measures they were taking, and if they knew that diabetes mellitus was a risk factor for COVID-19. Many measures specific for the assessment of quality of life in diabetic patients exist in scientific literature; 30 scales were identified [25]. Quality of life scales in diabetics consist of mental, physical, and social health components. Some diabetic quality of life scales also take into account the impact of public policies and societal attitudes [25]. The quality of life index from Carol E. Ferrans and Marjorie J. Powers (1998) [26] was used to assess the quality of life of diabetic patients. The questionnaire contains thirty-four items for the satisfaction of each of the four categories of life (health, the socio-economic domain of life, the psychological/spiritual domain of life, and the family domain) and thirty-four items for the importance of categories in quality of life. Questions about health and functioning issues were 1–8, 12, 17–19, 26, and 27; social and economic issues were examined by questions 14, 16, and 20–25; the psychological and spiritual domain questions were 28–34; and family issues in the home were examined by questions 9–11, 13, and 15. Answers to each question were, according to the Likert scale (1 = very dissatisfied/unimportant to 6 = very satisfied/important). The reliability coefficients of the Cronbach Alpha satisfaction scale before/after education were 0.957/0.969, and the importance scale before/after education was 0.968/0.976.

### 2.2. Ethical Consideration

The study was conducted in accordance with the ethical principles and human rights standards of medical study. For the purpose of conducting the investigation, the consent of the Commission for Ethical Affairs of the County General Hospital in Našice was obtained (Order No. 01-283/2-2019). The second focus was not the original aim of the study, but today, COVID-19 has become a crucial worldwide health problem, which is why we wanted to present our results to contribute to global knowledge about the quality of life of diabetics in the COVID-19 era. The third phase was only an extended study after the onset of the COVID-19 outbreak. No approval was required to amend or revise the questionnaires/interview questions (relating to COVID-19) used after the launch of the COVID-19 pandemic in Phase 3 in 2020. Subjects were contacted by telephone to attend a monitoring meeting where they completed the questionnaire (same) for the third time (and questions about COVID-19), measurements were repeated, and informed consent was signed to participate in an expanded study following the outbreak of COVID-19.

### 2.3. Statistical Analysis

To observe medium effect in the difference of continuous variables in the three measurements with a significance level of 0.05 and a power of 0.95, the minimum required sample size was 142 subjects (G * Power 3.1.9.2). Categorical data were represented by absolute and relative frequencies. Numerical data were described by the median and interquartile range in case of deviation from normal distribution (Shapiro—Wilk test). The Friedman’s test (Post hoc Conover) was used to detect the differences between dependent samples (before education, after education, during COVID-19 period). Effect size was reported as Cohen’s d [27]. Logistic regression for repeated measurements (GEE—Logistic Regression Model) was used to assess the impact of certain factors on the probability of a poorer assessment of the overall quality of life, as well as individual domains. We took the following independent variables into the model: gender, age, education level, place of residence, marital status, disease duration, BMI, and HbA1c. All *p* values were two-sided. The level of significance was set to Alpha = 0.05. The analysis was conducted while using the MedCalc Statistical Software version 19.4.1 (MedCalc Software Ltd., Ostend, Belgium; 2020) and the IBM SPSS Statistics 23 (IBM Corp. Released 2015. IBM SPSS Statistics for Windows, Version 23.0. IBM Corp., Armonk, NY, USA).

## 3. Results

### 3.1. Socio-Demographic Characteristics

A total of 205 participants took part in the study, of which 111 (54.1%) were men and 94 (46%) women. Seventy-three (36%) respondents were aged 51 to 60, or 61 to 70 years, while 36 (18%) were older than 70 years. Ninety-nine (49%) had secondary education, while 14 (7%) respondents had not completed primary school. One hundred and twenty-three (60%) respondents lived in rural areas, and according to marital status, 149 (73%) were married. Forty-six (22%) of them had minor children in the family, and 93 (45%) respondents had family members over the age of 60. Most respondents, 83 of them (41%) to be precise, had been diabetics for 2 to 5 years (Table 1).

### 3.2. Diabetes Health Parameters before and after Education and during the COVID-19 Period

There were no significant differences in the values of glucose, systolic, and diastolic pressure according to the measurements. HbA1c values were significantly higher before education compared to post-education at the time of COVID-19 (Friedman test, *p* = 0.002), as well as the mass of subjects (Friedman test, *p* < 0.001), while BMI was significantly lower after education compared to values before education (Friedman test, *p* = 0.008) (Table 2).

### 3.3. Quality of Life by Domains before and after Education and in the COVID-19 Period, and Differences Regarding Gender, Place of Residence and Age

The reliability coefficient of the satisfaction scale before/after the Cronbach Alpha education was 0.957/0.969 and the importance scale before/after the education was 0.968/0.976. There was a significantly lower quality of life in all domains during the COVID-19 period (Friedman test, *p* < 0.001), looking at the total and by gender. The assessment of the socio-economic domain and the psychological-spiritual domain does not differ significantly according to the measurements of the respondents living in urban areas. The overall assessment of socio-economic domains was significantly better assessed in the COVID-19 period, compared to before and after education (Friedman test, *p* < 0.001), and the significance is the same when looking at gender, age, and housing in rural areas. There were 112 (54.6%) respondents under the age of 62; we chose “age 62” as the cutoff, because 62 years was the median age of the respondents. Only in the case of assessing the quality of the psychological-spiritual domain were there no significant differences according to the measurements of respondents under the age of 62 (Table 3).

### 3.4. Self-Assessment of Behavior during the COVID-19 Pandemic

Forty-three (21%) respondents stated that COVID-19 is something they are not used to and 50 (24%) that COVID-19 is a real threat to people. Sixteen (8%) responders thought that this is just another type of the common flu, while 17 (8.3%) respondents stated that COVID-19 is a part of a worldwide conspiracy. Only 10 (5%) respondents believed that COVID-19 is God’s punishment, fabrication, and God’s will.

Due to the COVID-19 pandemic, 49 (36%) respondents stated that they followed the news more than they normally did; the same number of respondents said that they are very concerned about the COVID-19. Thirty-seven (27.4%) respondents stated that the virus is much more dangerous than the flu virus. Most of the respondents were convinced that they or a family member have a higher probability of contracting COVID-19 (Table 4).

Regarding precautionary measures outside the workplace, 22 (17%) respondents avoided physically approaching others, 40 (30%) avoided leaving their home, 32 (24%) washed their hands more than usual, 25 (19%) respondents bought more food than usual, 11 (8%) ingested a lot of vitamins, while three (2%) respondents stated that they tried to disinfect everything around them.

Only three (1.5%) respondents thought there are no high-risk diseases for COVID-19, while 12 (5.9%) did not know what they are. Fifty-six (27.3%) respondents stated diabetes is a high-risk disease for COVID-19, and 52 (25.4%) that lung diseases are high-risk. Obesity, a compromised immune system, internal organ diseases, and autoimmune diseases were mentioned to a lesser extent (Table 5).

### 3.5. Predictors of Worse Quality of Life in Patients with Type 2 Diabetes

Significant predictors of worse assessment of overall quality of life were male gender (OR = 3.69) and rural place of residence (OR = 3.14). Also, male gender was a significant predictor of worse health and functioning score (OR = 1.56). In the psychological/spiritual domain, significant predictors of worse assessment were male gender (OR = 3.11) and disease duration of up to 5 years (OR = 9.51). In the family domain, a significant predictor of a worse assessment was male gender, while being married (OR = 0.52) was a predictor that reduced the likelihood of a worse assessment for this domain. We did not find significant predictors that affect the likelihood of a worse socioeconomic domain score (Table 6).

## 4. Discussion

People suffering from diabetes feel the burden of their disease on a daily basis, because they need better control of a number of parameters, including fasting blood glucose, glycated hemoglobin, body mass index, and blood pressure (blood glucose, HbA1c, BMI, RR), which is achieved through organization and daily preference. However, in the COVID-19 era, it is even harder because of the pandemic and the lockdown. These parameters play a key role in regulating diabetes and preventing the development of complications. An important part of diabetes treatment is education that aims to improve the quality of life and life habits. Changing life habits may be the only therapeutic measure that does not depend on the type of diabetes. Based on this study, it was found that better educated patients have better control of blood glucose, HbA1c, and BMI. Gagliardino et al. conducted a training that included 59% of respondents and found that the control of HbA1c and blood glucose was 2.5 times better than the remaining respondents who did not undergo training [28]. A study in Italy demonstrated that BMI decreased over 5 years after periodically repeated education [29]. Research in Korea has shown also that the subjects had better HbA1c and blood glucose values after education [30]. Research conducted in Brazil concluded that education should be part of routine care for diabetics and should be repeated periodically, every eight to twelve months [31].

During the coronavirus pandemic, subjects had better blood glucose values than after the training program. Controlling their diabetes seemed to bring the belief that they could control an important part of their lives, even though they could not influence the pandemic and lockdown, which contributed to better mental quality of life. The Croatian healthcare system covered all costs of the educational program, and it was available to all. All subjects with diabetes who came to our diabetes clinic continuously had some kind of organized education (standard and non-standard). As other studies have shown, after periodically repeated education, subjects have better control of blood glucose, HbA1c, and BMI [29,31]. Poor HbA1c indicates low self-help behavior, increased barriers to daily life activities, and poor ability to positively manage diabetes, which causes poor quality of life and reduced health [32]. Improved diabetic control and better HbA1c is associated with improved mental, but not physical, quality of life over a one-year period in the community setting [33]. In this study, the coronavirus pandemic had a negative impact on the quality of life of diabetics, but the domain of psychological/spiritual quality of life was preserved, while the domain of health and functioning was reduced. It may also reflect the mental empowerment gained through proactive diabetes education and may reflect physical discomforts of increased regime complexity [33]. Enhancing self-care behaviors is essential to improve HbA1C control and quality of life [34].

Diabetic people could experience barriers during the COVID-19 period, such as limited access to healthcare, limited physical activity because of home confinement, and limited access to fresh food. The coronavirus pandemic negatively affected the body weight of the patients [22], which was not consistent with our study. In our study, the respondents maintained the same weight. Other research showed that, during a longer stay in the house and quarantine. there was a change in the daily routine, which caused an increase in the number of calories consumed from fast food in order to cope with stress [35]. To cope effectively with covibesity, change must happen. Future pandemic waves need careful contingency planning to reduce further escalation of covibesity. The public needs support with the help of health and education professionals, as well as changes in central government policy [17]. Protective factors for weight increase during COVID-19 confinement may be a healthy lifestyle that includes good food habits, physical activity, and active breaks [17]. As this study shows, lifestyle can be influenced by education.

Furthermore, Mostafa et al. concluded that after education, quality of life in the physical, social, and psychological domains would improve [36]. Patients would be satisfied with their health but also with their entire life. Patients’ quality of life decreased during the pandemic [37], which is consistent with our study. The sudden change in life habits marked a confrontation by the new situation, which was especially reflected in the field of health and functioning domain and family domain in this study. Many studies have concluded that women have a poorer quality of life than men, particularly in the areas of mental functioning, general health, social functioning, vitality, emotional, and mental health [36,37,38]. If we compare these data with our research, we can see that women showed greater satisfaction with the quality of life after the training. Compared to life and health satisfaction after the onset of COVID-19 pandemic, we can see that women expressed worse satisfaction than men. Married diabetic patients reported better quality of life in all subscales, and the differences were significant in general health, vitality, and mental health [39]. In this study, being married was a significant predictor of a better assessment of the quality of life in the family domain. The spread of the pandemic causes uncertainty and fear among the population. Fear and anxiety about a new disease and what could happen can be overwhelming and cause strong emotions in the population [40]. Being female, having diabetes complications, having changed diabetes behaviors, and feeling isolated and lonely, are associated with being more worried about COVID-19 and diabetes, which is associated with poorer psychosocial health [41]. Education should be based on the pre-test, targeting specific questions and worries regarding diabetes and COVID-19 in diabetics and on the new knowledge regarding COVID-19 and diabetes [41]. Our study can serve as a basis for preparing future education for diabetics, which will include questions about COVID-19. In this study, respondents believed that the coronavirus is more dangerous than the common flu and they were very worried about COVID-19, but less than half of the respondents believed that there is a possibility of infection, which indicated they know the dangers of COVID-19, but they think it is happening to someone else. In the review of the literature, Wicaksana et al. (2020) formed conclusions about specific recommendations for patients with diabetes during the coronavirus pandemic [42]. Diabetes patients, as a primary preventive method, should adhere to social distancing and home confinement policy. They should avoid contact with suspected or confirmed COVID-19 patients as much as they can. Online education is recommended, sharing free educational e-books and videos for diabetes management and COVID-19 prevention. It is recommended to consult through emails, phone calls, or video calls. Health care providers should always remind patients to wash their hands, wear a mask, and practice good cough technique and social distancing as general precautions. Advice for active physical activity while staying at home are stretching (e.g., yoga), muscle strengthening (e.g., light weightlifting), and aerobic exercise (e.g., dancing, cycling, treadmill, or sport aerobics). It is recommended that diabetics should arrange an individual plan for diabetes management while in home confinement and keep maintaining their glycemic control as part of risk-reduction of infection with COVID-19 [41]. Ruiz-Roso et al. recommended the development of public health policies to promote healthy lifestyles in terms of diet and physical activity, especially after this period of lockdown [43].

Han et al. stated the value of media during COVID-19. In their survey, more than 50% of respondents followed government instructions [44]. Our respondents did not follow the media significantly more than usual. Health communications via social media were positively significantly influenced by awareness and information exchange and indirectly influenced the adoption of preventive health care behavior [45]. The diabetes patients’ healthy lifestyle habits were significantly reduced after the lockdown [43,46]. The interaction between COVID-19 and diabetes mellitus sets up a vicious cycle wherein COVID-19 leads to worsening of glycemic control, and diabetes mellitus, in turn, exacerbates the severity of COVID-19 [47].

Taking into account the obtained results, it could be concluded that the coronavirus pandemic has a markedly negative impact on the quality of life of diabetics, but the domain of mental quality of life is preserved. Educational plans that improve or include strategies to enhance patient’s quality of life may increase compliance, thereby improving these patients’ diabetes parameters [48], which is then associated with improved quality of life [32,33,34], and a good educational plan includes all these parameters [40].

One of the limitations of this study was that we obtained data from a small geographical area, its findings could not be generalized to the entire Croatian diabetic population, and we did not include a control group. All subjects with diabetes in our clinic had some organized education (different from the standard of care) during treatment and follow-up, every 1 to 2 years, and only 25 (13%) subjects had a duration of diabetes for a year or less. Participants were recruited even if they had previously attended similar educational programs; consequently, we have only one group of respondents and no control group. We wanted to avoid “contamination” of the control group by intervention [49].

Future research directions may also be highlighted. Therefore, it is recommended to organize training classes, access resources and educational information, and facilitate access to [50] telemedicine and online education [42]. Further research is needed using a longitudinal study to understand how diabetes mellitus and the lockdown can affect the quality of life of diabetics and how quality of life changes during the pandemic. Joensen et al. (2020) concluded that further longitudinal studies are needed to explore if and how COVID-19 worries change during the pandemic in diabetics [41]. It would be ideal if the same psychometric tools could be translated. Validated, and used on a worldwide scale in order to explore differences in the populations and extract comparable results. Finally, diabetes is a strong and cunning enemy, demanding all of our resources, but technology development and the quality of the yet unexplored human brain provide us with the insinuation of a brighter dawn in the diabetes homeland [51].

## 5. Conclusions

This research shows that diabetes education is significantly correlated with effective HbA1c regulation, fasting blood glucose, BMI, body weight, and blood pressure. Education enhances knowledge and skills, but more importantly, it promotes positive attitudes towards active participation in the control and treatment of disease. A significant predictor of worse assessment of overall quality of life was male gender and rural place of residence. Male gender was a significant predictor of worse assessment of health and functioning, psychological/spiritual, and family domains. Disease duration of up to 5 years was a significant predictor of worse assessment in the psychological/spiritual domain, while being married was a predictor of better assessment of the quality of life in the family domain. We did not find significant predictors that affect the likelihood of a worse socioeconomic domain score. The coronavirus pandemic came abruptly and negatively affected the life habits of diabetics; however, we believe the results of measurements and their attitude would have been different if they had not undergone an educational program. It is not clear whether the levels of quality of life are attributable to the pandemic or to the effects of the educational intervention.

## Figures and Tables

**Table 1:** Baseline characteristics of the participants.

Sociodemographic Characteristics	[n (%)]
Gender [n (%)]	
Male	111 (54)
Female	94 (46)
Age [n (%)]	
up to 50 years	23 (11)
51–60 years	73 (36)
61–70 years	73 (36)
71 and older	36 (18)
Level of education [n (%)]	
Unfinished primary school	14 (7)
Elementary school	54 (26)
High school education	99 (49)
Higher education	37 (18)
Place of residence [n (%)]	
Rural	123 (60)
Urban	82 (40)
Marriage status [n (%)]	
Unmarried	19 (9)
Married	149 (73)
Divorced	7 (3)
Widower	29 (14)
Minor children in the family [n (%)]	46 (22)
Family members over the age of 60 [n (%)]	93 (45)
Duration of diabetes (years) [n (%)]	
Up to a year	25 (13)
2–5	83 (41)
6–10	50 (25)
11–15	23 (11)
≥16	21 (10)

**Table 2:** Diabetes health parameters before and after education and during the coronavirus disease 2019 (COVID-19) period.

Health Parameters	Median (Interquartile Range)			*p* *	Effect Size (Cohen’s d)		
	Before Education (1)	After Education (2)	COVID-19 Period (3)		1 vs. 2	1 vs. 3	2 vs. 3
Blood glucose [fasted]	7.3 (6.2–8.7)	7.1 (6.1–8.3)	7 (5.8–8.3)	0.34	0.01	0.07	0.11
HbA1c [%]	6.7 (6–7.7)	6.6 (6–7.3)	6.4 (5.7–7.1)	0.002 ^†^	0.19	0.04	0.08
Systolic pressure (mmHg)	130 (120–140)	130 (120–140)	130 (120–140)	0.54	0.10	0.12	0.03
Diastolic pressure (mmHg)	80 (75–90)	80 (75–90)	80 (80–90)	0.53	0.03	0.14	0.09
Weight (kg)	90 (78–102)	89 (76.8–100)	88 (75–102)	<0.001 ^†^	0.07	0.02	0.05
BMI (kg/m^2^)	31 (27.6–34)	30.8 (27.5–34)	31 (27.5–34)	0.008 ^‡^	0.06	0.02	0.03

HbA1c—glycated hemoglobin; BMI—body mass index * Friedman test (Post hoc Conover): ^†^ at the level of *p* < 0.05, there was a significant difference before vs. after, before vs. COVID-19; ^‡^ at the level of *p* < 0.05, there was a significant difference before vs. after.

**Table 3:** Quality of life by domains before/after education and in the COVID-19 period, and differences with regard to gender, place of residence and age.

Satisfaction with the Surrounding Life	Median (Interquartile Range)			*p* *	Effect Size (Cohen’s d)		
	Before Education (1)	After Education (2)	COVID-19 Period (3)		1 vs. 2	1 vs. 3	2 vs. 3
Health and functioning	21.3 (17.8–24.4)	21.5 (18.4–24.8)	13.4 (12.3–15.2)	<0.001 ^†^	0.06	2.12	2.19
Male	20.8 (17.7–24.0)	21.3 (18–24.4)	13.1 (11.9–14.5)	<0.001 ^†^	0.03	2.20	2.25
Female	22.3 (18.0–24.6)	22.4 (19.1–25.3)	14.4 (12.8–16)	<0.001 ^‡^	0.10	2.07	2.17
Rural	20.9 (17.5–24.0)	21.3 (18–24.4)	13.4 (12–15.2)	<0.001 ^†^	0.05	1.98	2.05
Urban	22.4 (18.0–24.8)	22.2 (18.6–25.7)	13.5 (12.4–15.5)	<0.001 ^†^	0.08	2.36	2.43
Age up to 62 years	21.8 (18.5–24.4)	21.9 (19.1–25.1)	13.4 (12.4–15.2)	<0.001 ^†^	0.08	2.32	2.35
≥62 years	20.8 (17.2–24.4)	20.7 (17.2–24.4)	13.4 (11.7–15.3)	<0.001 ^†^	0.04	1.93	2.02
Socioeconomic domain	21.6 (18–26)	22.1 (17.9–25.8)	23.3 (19.5–26.6)	<0.001 ^‡^	0.01	0.24	0.23
Male	21.3 (17.6–25)	21.93 (17.6–25)	22.7 (18.9–26.5)	<0.001 ^‡^	0.002	0.19	0.20
Female	22.1 (18.6–26.3)	22.3 (18.9–26.2)	23.9 (19.9–26.9)	<0.001 ^‡^	0.03	0.29	0.27
Rural	20.7 (17.5–24.6)	20.8 (17.6–24.5)	22.6 (18.7–26.3)	<0.001 ^‡^	0.004	0.31	0.33
Urban	23.6 (18.8–27.5)	23.9 (18.9–27.4)	24 (19.8–27)	0.91	0.04	0.14	0.10
Age up to 62 years	23 (18.4–26.3)	23.2 (18.9–26.2)	23.7 (19.9–27.8)	0.003 ^‡^	0.02	0.25	0.24
≥62 years	20.3 (17.2–25)	20.5 (17.2–25)	22.6 (16.7–25.5)	0.001 ^‡^	0.001	0.23	0.23
Psychological/spiritual domain	23.1 (19.2–26.6)	23.6 (19.3–27)	22.1 (18.2–25.5)	<0.001 ^‡^	0.009	0.20	0.21
Male	22.8 (17.6–26.7)	23.1 (17.7–26.4)	21.9 (17.9–25)	0.03 ^‡^	0.04	0.19	0.16
Female	23.9 (20–26.6)	24.3 (20.5–27.5)	22.5 (18.3–26.3)	<0.001 ^‡^	0.07	0.21	0.28
Rural	23.1 (18.6–26.3)	23.1 (18.7–26.1)	21.7 (17.8–24.2)	<0.001 ^‡^	0.03	0.29	0.26
Urban	23.9 (19.8–28.3)	24.3 (20.7–28.8)	24.3 (18.6–26.8)	0.05	0.07	0.07	0.15
Age up to 62 years	23.1 (18.5–27)	23.7 (18.9–27.7)	23.0 (20.0–26.1)	0.12	0.04	0.05	0.09
≥62 years	23.6 (19.7–26.6)	23.6 (19.8–26.1)	20.7 (17.2–25.0)	<0.001 ^‡^	0.04	0.39	0.36
Family domain	24.8 (21.4–28.5)	24.6 (21.7–28.5)	15.3 (13.2–17.3)	<0.001 ^‡^	0.04	2.33	2.42
Male	24.3 (21.4–28.8)	24 (21.2–28.3)	14.4 (12.9–16.8)	<0.001 ^‡^	0.02	2.43	2.44
Female	25.1 (21.3–27.7)	25.2 (22.2–28.5)	16.1 (13.8–18.3)	<0.001 ^‡^	0.11	2.24	2.45
Rural	24.6 (21.4–28.6)	24.1 (21.5–28.5)	15.1 (13–17.1)	<0.001 ^‡^	0.02	2.19	2.25
Urban	25.2 (21.3–28.2)	25.3 (22.3–28.5)	15.4 (13.2–17.6)	<0.001 ^‡^	0.08	2.58	2.76
Age up to 62 years (*n* = 112)	25.2 (21.6–28.8)	25.3 (21.6–28.8)	15.4 (13.5–17.3)	<0.001 ^‡^	0.06	2.30	2.43
≥62 years (*n* = 93)	24.6 (20.7–27.9)	24 (21.5–27.7)	15.2 (12.6–17.2)	<0.001^‡^	0.01	2.36	2.43
Quality of life	22.4 (18.6–25.4)	22.6 (18.8–25.6)	17.7 (15.8–19.8)	<0.001 ^†^	0.04	1.29	1.32
Male	22.2 (18.6–25)	22.2 (18.6–24.8)	17.3 (15.3–19.2)	<0.001 ^‡^	0.001	1.33	1.30
Female	22.71 (19–25.7)	23.1 (19.5–26.1)	18.2 (16.1–20.5)	<0.001 ^†^	0.09	1.27	1.37
Rural	22.1 (18.3–24.8)	22.3 (18.3–24.7)	17.6 (15.3–19.3)	<0.001 ^†^	0.02	1.24	1.23
Urban	22.9 (19.2–26.6)	23.5 (19.9–26.7)	18 (16.1–20.4)	<0.001 ^†^	0.08	1.40	1.49
Age up to 62 years	22.5 (18.9–25.8)	23.1 (19.5–25.9)	18.3 (16.0–20.2)	<0.001 ^‡^	0.06	1.28	1.32
≥62 years	21.9 (18.5–25.2)	21.9 (18.5–24.9)	17.6 (15.4–19.0)	<0.001 ^‡^	0.01	1.32	1.34

* Friedman test (Post hoc Conover): ^†^ at the level of *p* < 0.05, there was a significant difference before vs. after, before vs. COVID-19; after vs. COVID-19; ^‡,^ at the level of *p* < 0.05, there was a significant difference before vs. COVID-19, after vs. COVID-19.

**Table 4:** Self-assessment of behavior during the COVID-19 pandemic.

	Number (%) of Participants					
	Entirely Incorrect (0)	1	2	3	Entirely Exactly (4)	In Total
Because of the COVID-19 pandemic, I follow the news more than I normally do	12 (8.8)	10 (7.4)	24 (17.6)	41 (30.1)	49 (36)	136 (100)
	I do not think it is more dangerous than the flu	1	2	3	Much more dangerous from the flu	In total
To what extent do you believe that COVID-19 is more dangerous than the common flu?	9 (6.7)	10 (7.4)	24 (17.8)	55 (40.7)	37 (27.4)	135 (100)
	Not at all worried	1	2	3	Very worried	In total
How worried are you about the COVID-19?	8 (5.9)	17 (12.5)	20 (14.7)	42 (30.9)	49 (36)	136 (100)
	Not at all worried	1	2	3	Very worried	In total
What is the probability that you become infected with the COVID-19 virus?	8 (5.9)	18 (13.3)	36 (26.7)	48 (35.6)	25 (18.5)	135 (100)
How likely is it that a member of your family is infected with the COVID-19 virus?	11 (8.3)	21 (15.8)	37 (27.8)	46 (34.6)	18 (13.5)	133 (100)

**Table 5:** High-risk diseases for COVID-19.

	Number (%)
Diabetes mellitus	56 (27.3)
Lung diseases	52 (25.4)
Hypertension	25 (12.2)
Age	21 (10.2)
Heart disease	20 (9.8)
Any chronic disease	20 (9.8)
Malignant diseases	17 (8.3)
Asthma	7 (3.4)
Obesity	6 (2.9)
Compromised immune system	3 (1.5)
Internal organs diseases	2 (1)
Autoimmune diseases	1 (0.5)
There are no risky diseases	3 (1.5)
I do not know	12 (5.9)

**Table 6:** Significant predictors of worse quality of life in patients with type 2 diabetes.

	ß	Standard Error	Wald	OR	95%CI for ß	*p*
Quality of life						
Male	1.31	0.46	7.97	3.69	1.49 to 9.13	0.005
Rural	1.15	0.51	5.06	3.14	1.16 to 8.52	0.02
*Intercept*	−4.07	0.78	26.8	0.02	0.004 to 0.07	<0.001
Health and functioning						
Male	0.44	0.17	6.54	1.56	1.11 to 2.21	0.01
*Intercept*	−1.5	0.26	32.5	0.22	0.13 to 0.37	<0.001
Socioeconomic domain						
-	-	-	-	-	-	-
Psychological/spiritual domain						
Male	1.14	0.52	4.81	3.11	1.13 to 8.57	0.03
Duration of the disease (≤ 5 years)	2.25	1.04	4.61	9.51	1.22 to 74.31	0.03
*Intercept*	−4.98	1.26	15.5	0.007	0.001 to 0.08	<0.001
*Family domain*						
Male	0.89	0.23	14.3	2.43	1.53 to 3.85	<0.001
Marital status (married)	−0.65	0.32	4.29	0.52	0.28 to 0.96	0.04
*Intercept*	−1.75	0.29	34.8	0.17	0.09 to 0.31	<0.001

## Data Availability

The data presented in this study are available on request from the corresponding author.
